# Parental Reports of Preschoolers' Lexical and Syntactic Development: Validation of the CDI-III for European Portuguese

**DOI:** 10.3389/fpsyg.2021.677575

**Published:** 2021-07-22

**Authors:** Irene Cadime, Ana Lúcia Santos, Iolanda Ribeiro, Fernanda Leopoldina Viana

**Affiliations:** ^1^Psychology Research Center, School of Psychology, University of Minho, Braga, Portugal; ^2^Centro de Linguística da Universidade de Lisboa, Departamento de Linguística Geral e Românica, School of Arts and Humanities, University of Lisbon, Lisbon, Portugal; ^3^Research Centre on Child Studies, Institute of Education, University of Minho, Braga, Portugal

**Keywords:** MacArthur-Bates Communicative Development Inventory, vocabulary, syntax, validation, European Portuguese

## Abstract

This study presents the validation analysis of the European Portuguese version of the MacArthur-Bates Communicative Development Inventory III (CDI-III-PT). The CDI-III-PT is a parental report measure allowing researchers to assess expressive vocabulary and the syntactic abilities of children aged 2;6–4;0. In this study, we present a version comprising a lexical subscale which follows the Swedish adaptation and an original syntactic subscale allowing us to include language-specific structures. The reports of 739 children were collected; in addition, a standardized measure of language was also administered to a sub-sample of these children and the reports of preschool teachers were collected for another sub-sample. The results indicate a high internal consistency of the lexical and syntactic subscales. As for sociodemographic variables often found to be predictors of language development, as measured by this type of instrument, the results indicate that age and maternal education are significant predictors of the scores, and that first-born children attain higher scores in vocabulary than later born children, but no significant gender differences were found. The scores of the CDI-III-PT are positively correlated with the ones obtained in the standardized language measure, thus supporting their validity. A high agreement between the reports of parents and teachers was also found. These findings indicate that the CDI-III-PT has adequate psychometric properties and that it can be a useful tool for research and clinical practice. The age-based norms that are now provided can be used to evaluate whether a child is performing poorly compared to their peers.

## Introduction

The period after children's second year of life is usually associated with accelerated language development, both in terms of lexical knowledge and syntactic development. The vocabulary spurt—a rapid acceleration in the acquisition of new words—usually occurs around 18–24 months (Kauschke and Hofmeister, 2002; Chow et al., 2019). This phenomenon may be related to cognitive development and the resulting expansion of conceptual and categorization skills that occur at this stage (Pérez-Pereira, 2004). It is estimated that, at the age of 3 years, children have an expressive vocabulary of about 900–1,000 words and can use up to 12,000 individual words per day (Owens, 2012). At this stage, the lexicon is varied and includes a large portion of nouns, as well as verbs, adjectives, and function words (Kauschke and Hofmeister, 2002). Around the fourth birthday, children's expressive vocabulary can consist of ~1,500 words, and 1 year later, the number of new words acquired can exceed 500 (Owens, 2012). This increase in vocabulary size is accompanied by a higher prevalence of more diversified types of words, such as mental state words. Mental state verbs like *know* and *think* are produced by children younger than 3 years of age, although they may not have adult-like comprehension of the sentences in which these verbs occur (Shatz et al., 1983; Bloom et al., 1989; Lewis et al., 2017; also Hacquard, 2014, on syntactic bootstrapping of the meaning of attitude verbs). Along with children's cognitive development and as they are more exposed to mental state words, these types of words start to be more incorporated in their lexicon (de Villiers, 2000; Harris et al., 2005). For example, in a study by Nixon (2005) with 4-year-old children, the results indicated that they produced, on average, 14 tokens and 5 types of mental state verbs in a 15-min task of interaction with a caregiver. Importantly, mental state verbs take clauses as their complements and the emergence of complement clauses is one of the signs of the rapid syntactic development in this age period.

The third year of life is typically characterized by both a relevant increase in the extension of children's utterances and a clear increase in the syntactic complexity of these utterances, resulting from expansion of the structures that can be attested in children's spontaneous production. This increase in complexity typically corresponds at this stage to the emergence of different syntactic structures, namely, complement clauses with an overt complementizer, relative clauses, clefts, some adverbial subordinate clauses, as well diverse types of wh-questions. The emergence of this cluster of structures in spontaneous production is documented in work based on corpora of European Portuguese (see Soares, 2006; Santos, 2009; Lobo et al., 2016). Moreover, in generative syntax, the emergence of these structures as a cluster can be associated with the fact that these structures have in common the activation of the C(omplementizer) P(hrase) domain, a higher layer of syntactic structure hosting complementizers, relative pronouns, and wh-phrases in the left periphery of the clause or clefted constituents. The need to explain the syntactic development observable in spontaneous production in the first years of life has justified a long tradition in the generative literature of discussing the presence of functional projections in the first stages of language production (see Radford, 1988; Rizzi, 1993), which has been resumed in the recent proposal of the Growing Trees Hypothesis (Friedmann et al., 2020). It is not the aim of this study to take a position in this theoretical debate; instead, we take, as a departure point for the definition of a syntactic scale of development in this age period, the solid descriptive basis of the discussion, which documents the emergence of a cluster of structures which can be associated with the activation of CP, roughly after the second year of life.

Finally, despite the general homogeneous trends in development observed in children during their early development, some studies have suggested that different sociodemographic factors may contribute to explain a more accelerated linguistic development, especially in the case of lexical development, and consequently children's scores in different instruments measuring early language development.

It is the aim of this paper to present a European Portuguese version of the MacArthur-Bates Communicative Development Inventory III (CDI-III-PT) for children between 2;6 and 4;0 (Fenson et al., 2007 for the original version). This adaptation follows previous successful adaptations in the case of the scale measuring lexical development, but departs from previous adaptations in the case of the scale measuring syntactic development. In what follows, we start by presenting a summary of what the literature based on spontaneous speech data suggests to be the syntactic structures that emerge in the third year of life. In doing so, we particularly explore the specificities of the syntax of European Portuguese and justify our choices for the new syntactic scale; we also explore the relations between the emergence of these syntactic structures and lexical development, aiming to show the interdependence of certain aspects of syntactic and lexical development. We continue summarizing what the literature has shown to be sociodemographic factors contributing to explain the acceleration in certain aspects of language development, and which are factors that we should consider in the validation study. We end this introductory section by presenting information on the original version of the CDI-III (Fenson et al., 2007), as well as on its very few adaptations, especially the Swedish adaptation, which we followed when creating the lexical subscale of the Portuguese adaptation. In the second part of the paper, we present the goals, the method, and the results of the present study, allowing us to validate the CDI-III as a new instrument available for the assessment of the European Portuguese population. All the materials allowing the use of the instrument are presented as Appendices to the present paper and will therefore be freely available.

### The Emergence of Complex Syntax After Age 2 and the Specificities of European Portuguese

The third year of life corresponds to a period of rapid development manifested in the extension of children's utterances and, simultaneously, in the syntactic complexity of these utterances. The adaptation of the CDI-III to European Portuguese, which we present in this paper, was designed to target the main syntactic developmental markers in this age period. In this section, we identify the markers of syntactic development that we considered in the present study.

A relevant set of structures identified in the spontaneous speech of children in this age range, but absent in previous periods, corresponds to certain wh-questions—associated with the production of a larger set of interrogative pronouns (e.g., *quem* “who,” *o que* “what”) and interrogative adverbs (*onde* “where”, *como* “how”)—relative clauses, cleft structures, and finite complement clauses—associated with the presence of a complementizer (*que* “that”), as well as certain adverbial subordinate clauses—associated with the presence of conjunctions with different semantic values.

On the basis of Soares (2006), it is possible to assert that wh-questions emerge in children's spontaneous production before 2;00, but are restricted to certain wh-elements (*o que* “what,” *onde* “where,” followed by *quem* “who”) typically combined with copula verbs. However, a very common type of wh-questions in European Portuguese is built with *é que* following the wh-expression—this “*é que*” has been analyzed as a frozen expression which is re-analyzed as a lexical unit filling the head of CP in generative syntactic analyses. According to Soares (2006), *é que* first emerges with the wh-word *onde* “where” between 2;6 and 3;2 (as in 1). We take this *é que* wh-question as a relevant marker of syntactic development in this age period.


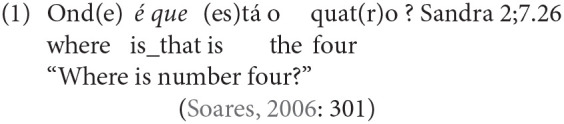


Other related structures which typically emerge in children's speech after 2 years of age are relative clauses and clefts. Relative clauses in child speech include object relatives, but are non-reversible, therefore not of the type of object relatives which typically justify difficulties in comprehension and production (see Friedmann et al., 2009; Costa et al., 2011 for European Portuguese). As for clefts, Lobo et al. (2016) show that clefts start to be produced between 2;01 and 2;03. The most productive types of clefts are standard clefts and *é que* clefts, the latter is a type of cleft specific to European Portuguese, in which we again find a lexicalized *é que* expression following the clefted material [see (2)]. On the contrary, pseudoclefts—and in general clefts involving the presence of a wh-expression—are rare in child speech at this age range. Subject clefts are generally more frequent than object clefts.


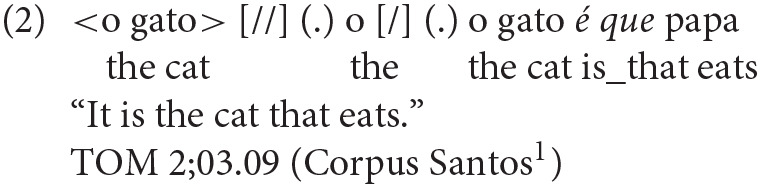


Another marker of syntactic development is the growth in frequency and in diversity of the complement clauses produced by the child, associated with the acquisition of verbs taking clauses as complements. In this case, very early occurrences of *querer* “want” with an infinitival complement clause are found in spontaneous speech before 2;00 (Santos, 2009). However, infinitival complement clauses under object control verbs (such as *ensinar* “teach” or *ajudar* “help”) and subject control verbs of the type of *prometer* “promise” have been shown to cause difficulties in comprehension (Agostinho et al., 2018), as well as in production (Santos et al., 2016b; Santos, in press). These difficulties are salient until at least 4 years in the case of object control structures and until later in the case of subject control with *prometer* “promise” (see Chomsky, 1969 for the prolonged difficulty in the comprehension of control structures with *promise*).

Other types of infinitival complements occur under perception verbs and causative verbs. In the case of perception verbs, Santos et al. (2016b) document high frequency at 3 years of age of the perception verb *ver* “see” taking a Prepositional Infinitival Construction (PIC) as a complement. This structure is specific to Portuguese and associated with the presence of an aspectual marker *a*. The same study suggests that structures with causative verbs (namely *deixar* “let”) may be more difficult for 3-year-olds, with a high proportion of the occurrences of this verb in spontaneous production reduced to the same word combination *deixa-me* + *infinitive* “let me + infinitive.”

Important markers of syntactic development during the third year of life are finite complement clauses. The first finite complement clauses are indicative clauses under the verbs *achar* “think” and *dizer* “tell,” and are preceded by the earlier occurrence of simpler verbless complement clauses consisting of a complementizer *que* “that” and a polarity adverb (see Santos, 2009). Soares (2006) shows that finite complement clauses present a low frequency until 3;6.

In general, the verb *querer* “want” precedes *achar* “think” in children's spontaneous speech, mirroring the contrast between *want* and *think* in English (see Hacquard, 2014). However, *querer* “want” emerges early in child speech but only in structures in which it takes an NP or an infinitival clause as a complement. With a finite complement, *querer* “want” selects a subjunctive complement and, even though this is the first type of subjunctive complement clause spontaneously produced by children, it occurs at a more advanced period, around 3 years (Jesus, 2014; Jesus et al., 2019)—in general, the subjunctive in complement clauses shows a protracted development (e.g., Blake, 1983).

Finally, it has been suggested that certain connective words associated with adverbial subordinate clauses emerge in this period in development: *se* “if” first emerges in the Santos corpus between 2;5 and 2;9 (Costa et al., 2008), and is preceded by *quando* “when” (see also Diessel, 2004 on English connectives).

Considering the set of structures that we have assumed to characterize language development starting at 2;00, we identify a cluster of structures in which subordination plays a salient role, as well as other structures which the generative tradition associates with the activation of a Complementizer Phrase layer (this is the case of wh-questions). It is also clear the extent to which lexical and syntactic development are connected: the acquisition of different types of complement clauses is dependent on the acquisition of different verbs; the acquisition of wh-questions is also defined by the set of interrogative pronouns and adverbs that the child produces; the acquisition of connective words, including complementizers, is directly related to the development of different types of subordination.

### Sociodemographic Factors Frequently Associated With Language Development

Several demographic factors have been associated with children's language development. One of the most studied factors is gender. Several studies with infants and toddlers have found gender differences, favoring girls, in language development (Bauer et al., 2002; Bleses et al., 2008; Bouchard et al., 2009; Eriksson et al., 2011; Trudeau and Sutton, 2011; Simonsen et al., 2014; Silva et al., 2017b). However, some studies with toddlers suggest that, as they approach 3;0, there is a tendency for boys to catch up in terms of expressive language (Simonsen et al., 2014). The results of other studies with preschool children, with ages ranging approximately between 3;0 and 6;0, seem to confirm that this gender gap decreases as children grow older: some studies with preschoolers show only small differences favoring girls (Jin et al., 2020; van der Wilt et al., 2020), and others do not show any differences (Altinkaynak, 2019; Fung et al., 2019).

Another factor that has been consistently related to children's linguistic skills is maternal education. Several studies have found evidence that a higher level of maternal education is associated with more developed linguistic skills in children, both in toddlers and in preschool children (Umek et al., 2006; Weigel et al., 2006; Fenson et al., 2007; Richels et al., 2013; Andonova, 2015; Gonzalez et al., 2017; Cadime et al., 2018; Farley and Piasta, 2020). Research has also consistently shown that mothers with higher educational levels provide more linguistic input, use a more varied vocabulary and more complex syntax, and more frequently perform activities such as reading for their children, than mothers with lower educational levels (Hoff, 2006; Gonzalez et al., 2017; Sultana et al., 2020), which may explain the consistent relationship that has been found between maternal education and children's language abilities. Regardless of these findings, some studies, mostly conducted with children up to 2 years old, did not find significant differences as a function of maternal education (Stolt et al., 2007; Gendler-Shalev and Dromi, 2021).

Another variable that seems to be associated with language development is birth order. The results of some studies with toddlers suggest that first born children have better lexical and grammatical skills than later born children (Fenson et al., 1994; Hoff-Ginsberg, 1998; Berglund et al., 2005; Zambrana et al., 2012). This relationship has been explained by the variations in the access to communicative interaction with adults, as first born have more interactions and more exposure to child-directed speech from adults than later born children (Hoff, 2006).

However, studies that used different types of measures to assess children's language abilities have found inconsistent results regarding birth order effects: the effect was verified when using parental reports but not when using direct observations of children's spontaneous speech (Jones and Adamson, 1987; Bornstein et al., 2004).

### Previous Adaptations of the MacArthur-Bates Communicative Development Inventories (and the Particular Case of the CDI-III)

The MacArthur-Bates Communicative Development Inventories (CDI) are measures of the communicative development of young children. The CDI, which includes different versions for different age stages, is based on the parental reports of the children's abilities (Fenson et al., 2007). The CDI—Words and Gestures and the CDI—Words and Sentences, for children between 0;8 and 2;6, have been adapted for dozens of languages. The adaptation studies for the different languages, including European Portuguese, have provided strong evidence of validity and reliability for these versions (e.g., Feldman et al., 2000; Bleses et al., 2008; O'Toole and Fletcher, 2010; Pérez-Pereira and Resches, 2011; Simonsen et al., 2014; Silva et al., 2017a,b).

The CDI-III is the version designed to assess lexical and syntactic development of children older than 30 months. In contrast with the CDI versions used to assess younger children, this version has not yet been adapted to many languages (exceptions are the adaptations to Basque, Swedish, and Estonian, which are already published, and which we briefly discuss in this section). Notwithstanding, the interest of adapting this type of measure for children at 2;6 and older is clear: it coincides with the moment at which children enter their pre-school years and the moment when parents and educators often manifest their first worries concerning language production skills.

The original version of the CDI-III (Fenson et al., 2007) was developed in the USA to assess children between 30 and 36 months old. It is composed of three subscales: vocabulary (100 items), syntactic complexity (12 items), and uses of language (12 items). This version was validated using a normative sample of 356 reports. The validation study suggested the presence of severe ceiling effects in the subscales “Syntactic complexity” and “Uses of language,” especially after 33 months.

There are some adaptations of the CDI-III for other languages, but most of them differ in structure and target population. In 2014, Garcia et al. (2014) adapted the CDI-III for Basque. The adaptation maintained the same subscales as the American version, but a morphology subscale was added. This version was validated using a sample of 1,024 children with ages ranging between 2;6 and 4;2. However, Garcia et al. (2014) found ceiling effects after 3;6 in all subscales.

The adaptation for Swedish (Eriksson, 2017) comprises three subscales: vocabulary (100 items), syntax (18 items), and metalinguistic awareness (7 items). It also includes one item aiming to assess children's level of communication, which serves as a first filter (if the child does not speak yet or his/her speech is not intelligible, no further questions are asked), and one item more generally assessing phonological development (parents should indicate if the child sounds like a typical child of that age or not). The main difference regarding the other CDI-III versions is related to the vocabulary section. The 100 words included in the checklist are divided into four semantic categories: food words (16 items), body words (26 items), mental words (30 items), and emotion words (28 items). The syntax section is divided into two sections: (i) language complexity, a section presenting pairs of sentences corresponding to different complexity levels, which should be chosen by parents according to the degree of similarity to the child's typical utterances; and (ii) grammar, where parents should indicate if their child uses particular constructions such as past tense, modifiers, or connective words. The metalinguistic awareness section includes items which assess phonological awareness, orthographic awareness, and awareness of the existence of other languages. The Swedish CDI was validated with 1,134 children aged between 2;6 and 4;0. Adequate psychometric properties were found; namely, evidence of validity based on the internal structure, high internal consistency of the items, and high intercorrelations between the subscales. The results of the validation study indicate a high percentage of variance explained by age (around 20%) but a slight ceiling effect was also found in the syntactic complexity and metalinguistic awareness subscales after 3;9 (45 months).

More recently, an Estonian adaptation was developed (Tulviste and Schults, 2020). This version was based on the Swedish version and presents a similar structure, albeit with some variation in the number of items of each subscale. The results of the validation study (Tulviste and Schults, 2020), in which the reports of 100 Estonian-speaking children aged between 2;10 and 3;3 were analyzed, indicated high levels of internal consistency for the vocabulary and syntax subscales, but low levels for the metalinguistic awareness subscales, probably due to the low number of items. Regarding validity evidence, medium-to-large intercorrelations between the subscales of the CDI-III and medium correlations between the scores in the CDI-III and other standardized measures were found. The results of the study also indicated that being a girl, having a higher number of older siblings, having attended day care for a longer time, having a mother with higher educational levels, and having no reported language difficulties are factors associated with a higher score in the vocabulary subscale (Tulviste and Schults, 2020). The effect of these demographic variables on the remaining CDI-III subscales was not tested.

To conclude, there are a few adaptations of the CDI-III for different languages with varying formats and psychometric properties. As can be seen in the overview presented in Table 1, the two most recent adaptations (for Swedish and Estonian) are more similar, whereas the original version and the Basque adaptation had more communalities. We can conclude from our review that the most recent ones have also demonstrated more robust psychometric properties, especially regarding the vocabulary subscale.

**Table 1:** Overview of the CDI-III versions.

**References**	**Language**	**Age range of the target population**	**Subscales and items**				
			**Vocabulary**	**Syntax and morphology**	**Uses of language**	**Metalinguistic awareness**	**Additional items**
Fenson et al. (2007)	English (American)	2;6–3;0	(a) Word checklist (100)	(a) Syntactic complexity (12)—Pairs of sentences with varying complexity (parents must flag one in each pair that most resembles what the child says)	(a) Using language (12)—Questions on different language uses with a yes/no response	–	(a) One question on whether the child already combines words (not yet/sometimes/often) (b) Mean length of utterances—Parents must list the three longest sentences that they heard from their child recently
Garcia et al. (2014)	Basque	2;6–4;2	(a) Word checklist (120)	(a) Syntactic complexity (29)—Pairs of sentences with varying complexity (parents must flag one in each pair that most resembles what the child says)(b) Morphology—one list of suffixes (16) and one list of verbs (20) (parents should indicate the ones produced by their child)	(a) Using language (10)—Questions on different language uses with a yes/no response	–	(a) One question on whether the child already combines words (not yet/sometimes/often) (b) Mean length of utterances—Parents must list the three longest sentences that they heard from their child recently
Eriksson (2017)	Swedish	2;6–4;0	(a) Word checklist (100) divided into four semantic categories: food words (16), body words (26), mental words (30), emotion words (28)	(a) Language complexity (10)—Pairs of sentences (parents must indicate if the child uses one of them or if both are used equally)(b) Grammar (8)—questions on the use of grammar markers with a three-category frequency response scale: never/sometimes/everyday	–	(a) Metalinguistic awareness (7)—Questions with a yes/no response to assess phonological and orthographic awareness, as well as the awareness of the existence of other languages	(a) One question on children's general level of communication with six alternatives (e.g., not yet talking) (b) Pronunciation—one question on how the child sounds compared to other children of the same age, with three response alternatives: like an age-mate, a younger, or an older child.
Tulviste and Schults (2020)	Estonian	2;6–4;0^*^	(a) Word checklist (101) divided into four semantic categories: food words (17), body words (26), mental words (30), emotion words (28)	(a) Language complexity (10)—Pairs of sentences (parents must indicate if the child uses one of them or if both are used alternately)(b) Grammar (7)—questions on the use of grammar markers with a three-category frequency response scale: never/sometimes/everyday	–	(a) Metalinguistic awareness (7)—Questions with a yes/no response to assess phonological and orthographic awareness	(a) One question on children's general level of communication with six alternatives (e.g., not yet talking) (b) Pronunciation—one question on how the child sounds compared to other children of the same age with three response alternatives: like an age-mate, a younger, or an older child. Five items with a yes/no response to assess specific pronunciation difficulties.

*The numbers presented within brackets are the number of items in each subscale*.

* *For the Estonian version, so far only a study with children aged between 2;10 and 3.3 was published (Tulviste and Schults, 2020). The norming study for the age range 2;6-4;0 is ongoing*.

### The Present Study

The main goal of this paper is to present the results of the validation studies of the European Portuguese adaptation of the CDI-III (CDI-III-PT). In order to achieve this main goal, specific goals were defined, namely:

1. To examine the internal consistency of the scores in the two CDI-III-PT subscales;
2. To determine whether the CDI-III-PT scores can capture the variation in vocabulary and syntax that are expected across the age range 2;6–4;0;
3. To collect evidence of validity based on the relations to other variables [according to recommendations of the American Educational Research Association (AERA) et al., 1999], by: (i) investigating the extent to which the child's gender, maternal education, and birth order account for variability in vocabulary and syntax, as measured by CDI-III-PT (validity based on test-criterion relationships); and (ii) exploring the relationships among the scores in the CDI-III-PT subscales and the scores in a pre-existent standardized measure of children's language abilities (convergent validity);
4. To analyze the correlation and agreement between the scores provided by parents and preschool teachers in the CDI-III-PT.

Convergent validity is based on the premise that the test scores should be related to the ones of other measures intended to measure similar constructs [American Educational Research Association (AERA) et al., 1999]. For the purposes of this study, the language subscale of the Griffiths Mental Development Scale was used as a reference measure. The reasons for this choice were twofold: (a) it is a standardized language measure that covers the full targeted age range (2;6–4;0) and is available in European Portuguese; and (b) it is more reliable and easier to administer and score than other non-standardized measures of language (e.g., spontaneous speech measures). This subscale provides a composite score that comprises receptive and expressive language abilities, as well as syntactic, semantic, and pragmatic abilities.

Nowadays, many children attend regular care outside their family. This is especially true in Portugal, where around 40% of children up to 3 years old and around 90% of the children aged between 3 and 5 years old attend daycare or preschool institutions (OECD, 2020). Thus, similarly to parents, daycare and preschool teachers are key observers of the children's linguistic productions. The correlation and agreement between different caregivers are important indicators of the reliability of the scores. Previous studies investigating the agreement between parents' and teachers' scores in expressive language measures indicate that the agreement between those is high and comparable to mother-father ratings (Stolarova et al., 2014). Moreover, the correlations between the scores of parents and teachers in measures of children's development—which indicate that the informants rank children similarly—are usually medium to high (e.g., Koch et al., 2011; Stolarova et al., 2014). High correlations (>0.50) between the teachers' and parents' scores in the European Portuguese versions of the CDI—Words and Gestures and of the CDI—Words and Sentences have also been reported (Viana et al., 2017). Therefore, similar results are expected for the CDI-III-PT.

## Methods

### Participants

The target population was children aged between 2;6 and 4;0. We established, as a minimum, 15 children per month of age for data collection and the following exclusion criteria were defined: children born prematurely with low weight (<32 weeks of pregnancy and <1,500 g), children whose mother and father did not speak European Portuguese, and children with severe medical conditions that could result in language impairment (e.g., Down syndrome). The reports of 795 children were collected. The data of 56 children were excluded from the initial sample either because they were not inside the age range or because they fell into one of the exclusion criteria. Thus, the final sample was composed of 739 children (mean age = 38.51 months old; SD = 4.72), who attended 56 different preschool education institutions. Table 2 presents the demographic characteristics of the sample (note: the final *N* for each age month can be consulted in Appendix C). Birth order ranged between the first and sixth child, with most of the children (55.2%) being a first-born child. The proportion of boys and girls was similar across all months of age, $\chi_{\left(18\right)}^{2}$ = 17.498, *p* = 0.489. The proportion of children from mothers with higher education degrees and from mothers with lower degrees was also similar across the age groups, $\chi_{\left(18\right)}^{2}$ = 23.984, *p* = 0.156.

**Table 2:** Characteristics of the participants.

	***N***	**%**
Gender		
Girls	337	45.6
Boys	397	53.7
No information	5	0.7
Birth order		
First born child	408	55.2
Later born child	303	41.0
No information	28	3.8
Mother education		
Secondary education or less	343	46.4
Higher education	391	52.9
No information	5	0.7

The preschool teachers of two classes (one in the Northern region and another in the region of Lisbon) were asked to fill in the CDI-III. Thus, for 23 children of the sample, information was collected from the parents and from the teachers. Another sub-sample composed of 23 children from one institution located in the North of Portugal was administered the language subscale of the Griffiths Mental Development Scale.

### Measures

#### MacArthur-Bates Communicative Development Inventory III—Adaptation for European Portuguese

A first version of the CDI-III in European Portuguese was developed, composed of two subscales: vocabulary and syntax. In the vocabulary subscale, a word checklist is presented, and the parents are asked to flag the words that their children produce spontaneously. In terms of structure, the vocabulary checklist is similar to the one of the Swedish CDI-III version (Eriksson, 2017), being divided into four lexical categories: (1) body parts and related words; (2) food and related words; (3) mental terms; and (4) emotions and related words. The choice of the lexical items to be included in the list was not only based on the Swedish CDI-III, but mainly on the Portuguese version of the CDI—Words and Sentences for toddlers aged between 1;4 and 2;6 (Silva et al., 2017b) and on a lexicon of European Portuguese child and child-directed spontaneous speech (Santos et al., 2016a). This first version included 117 words.

The syntax subscale is original and composed of a 26-item checklist presenting examples of syntactic structures. For each item, the parents must indicate (yes/no) if the child produces the target structure. The target structure is made salient by the presentation of one or more example sentences with relevant words in bold (for example, in the case of wh-questions, the wh-word, the expression *é que* or both, as well as the punctuation mark signaling that the utterance was a question, could occur in bold). This checklist focuses structures that are expected to emerge at the relevant age period, according to the description presented in the introduction section of this paper. This subscale includes the following structures:

(i) *Querer* “want” with an NP complement (one item).
(ii) Wh-questions (5 items). One item corresponds to simpler structures combining *o que/que* “what” with a copula verb. The remaining items are *é que* wh-questions with different wh-words (*onde* “where,” *quem* “who,” *porque* “why,” and *como* “how”).
(iii) Relative clauses (2 items). One item presents relative clauses with *que* “that;” one item presents a relative clause introduced by *onde* “where.”
(iv) Clefts (3 items). One item corresponds to an *é que* cleft, a second item to a standard cleft and the third item is a pseudocleft. All are cases of subject clefts.
(v) Infinitival structure under a modal verb (1 item).
(vi) Infinitival complements (8 items). These include subject control structures under transitive verbs (*querer* “want” and *gostar* “like”), the subject control structure under the ditransitive verb *prometer* “promise,” and object control structures. They also include the Prepositional Infinitival Construction under *ver* “see,” infinitival complements of *deixar* “let,” including the most frequent form in spontaneous speech *deixa-me* + *infinitive* “let me + infinitive.”
(vii) Complements with an overt complementizer *que* “that” (4 items). One item corresponds to the simpler form with an embedded polarity adverb found in early stages in spontaneous speech [*acho que sim/não* “I think it is (not) the case.”]. Two other items correspond to complement clauses taking indicative, under *achar* “think” or *dizer* “say.” A fourth item corresponded to a subjunctive complement clause under *querer* “want.”
(viii) Adverbial clauses (2 items). One item corresponds to a temporal clause introduced by *quando* “when;” the second item is a conditional introduced by *se* “if.”

A pilot study for this first version of the CDI-III-PT was conducted with 88 children, aged 2;6–4;0. The results of this pilot study suggested a slight ceiling effect in the vocabulary subscale and a low correlation of the scores in this subscale with age. Therefore, the checklist was revised: the items with very high frequencies (>90%) and low correlation with age were deleted and new items were added. No revision of the syntactic subscale was considered relevant. Therefore, the final version of the CDI-III-PT, used in this study, is composed of a 166-item vocabulary checklist (body parts and related words: 34 words; food and related words: 37 words; mental terms: 45 words; emotions and related words: 50 words) and a 26-item subscale to assess syntactic complexity. These materials are available in Appendix A.

#### The Griffiths Mental Development Scales—Extended and Revised

This scale (Luiz et al., 2008) is administered individually and assesses the overall development of children aged 2–8 years old. It consists of six subscales: Subscale A (locomotion), Subscale B (personal-social), Subscale C (language), Subscale D (eye-hand coordination), Subscale E (realization), and Subscale F (practical reasoning). In this study, only subscale C was used, which assesses the child's receptive and expressive language, that is, it evaluates the child's ability to perform tasks such as naming objects and colors, repeating sentences, describing an image, and answering questions of comprehension, similarities, and differences. This subscale contains 38 items, which are rated as success or failure. Raw scores are then converted into *z*-scores.

#### Sociodemographic Questionnaire

Parents who filled in the CDI-III-PT were also asked to fill in a sociodemographic questionnaire to collect information such as the parents' education, child's age, gender, birth order, prematurity, or presence of clinical conditions.

### Procedure

Authorization for data collection was obtained from the National Commission of Data Protection, thus guaranteeing that legal and ethical issues for data collection were considered. Data collection was performed with the collaboration of daycare/preschool institutions. The selection of daycare/preschool institutions was made on a convenience basis: institutions that had already collaborated in previous projects of the research team were contacted successively until the required number of participants was achieved. The preschool teachers of the institutions that agreed to participate contacted the parents of the eligible children, explained to them the goals of the study, and distributed paper copies of the informed consent. Paper copies of the CDI-III-PT and the sociodemographic questionnaire were then delivered to parents who returned the signed informed consent. Parents returned the filled instruments in a closed envelope. The majority of the inventories (88.5%) were filled by the mother and the remaining by the father of the child, mother and father together, or another main caregiver. The Griffiths Mental Development Scale was administered to the previously referred sub-sample, by a trained psychologist, in a quiet room in the institution attended by the children, following the instructions and standardized procedures of the respective manual. For the second sub-sample (see participants' description), the CDI-III-PT was filled both by parents and preschool teachers. In this case, a unique code was attributed to each child and printed in the protocols to allow the pairing of the information collected from the different informants.

### Statistical Analysis

Reliability of the vocabulary and syntax subscales was analyzed by means of the Kuder-Richardson Formula 20 (KR-20). A minimum value of 0.70 was considered an indicator of an adequate internal consistency. Next, descriptive statistics of the CDI-III-PT were computed. Skewness and kurtosis values were analyzed to determine violations to the normality of the distributions, with values close to zero indicating no robust violations (Field, 2009). Pearson correlation coefficients were used to determine the intercorrelations among the CDI-III-PT indicators and to explore the relationship between the scores obtained in the CDI-III-PT and another measure of language (Griffiths Mental Development Scale—Language subscale). The same statistical analysis was used to compute the relationship between the scores provided by parents and preschool teachers. Cohen's guidelines were used to analyze the size of the correlations: 0.10 indicates a small effect, 0.30 a medium effect, and 0.50 a large effect (Cohen, 1992). Paired samples *t*-tests were computed to test for differences in the mean scores provided by parents and preschool teachers. Cohen's *d* was used as a measure of effect size for these analyses: values higher than 0.20 represent a small effect, higher than 0.50 a medium effect, and higher than 0.80 a large effect (Cohen, 1992). Multilevel modeling was used to analyze the effects of age, gender, maternal education, and birth order on vocabulary and syntax, as measured by the CDI-III-PT. This type of analyses was used to account for the clustered structure of the data, given that the data were collected in 56 different preschool institutions. Maximum likelihood estimation was used. A fully unconditional model for each dependent variable was firstly tested to serve as a baseline model and, in a second step, the predictors were added. Mean centered values were used for age, as it is a continuous predictor. A linear relationship between age and the dependent variables was also verified, as this is one assumption for this type of analyses. The log-likelihood ratio test was calculated to explore the difference in the fit of the baseline models and the models with predictors. These analyses were computed using IBM SPSS Statistics 25. Percentiles and growth charts were based on fitted values. Scores were fitted using a linear model, given that more complex growth models (quadratic and cubic), did not show a superior fit in our data (see Table A and Figures A,B in Appendix B for model comparison). The R package quantregGrowth (Muggeo et al., 2013; Muggeo, 2018) was used to obtain these fitted values and to construct the charts.

## Results

### Internal Consistency and Descriptive Statistics

KR-20 was 0.981 for the vocabulary subscale and 0.911 for the syntax subscale. Table 3 presents the descriptive statistics of the scores in both CDI-III subscales. Regarding the vocabulary subscale, 3 children (0.4%) did not produce any words from the body parts section, 8 (1.1%) from the food section, 14 (1.9%) from the mental terms section, and 3 (0.4%) from the emotions section. However, all children produced some of the words of the total list (minimum = 3; maximum = 166). For two children (0.3%), parents indicated that they did not produce any of the syntactic structures of the second subscale. The skewness and kurtosis were low, suggesting no robust violations to the normality of the distributions.

**Table 3:** Descriptive statistics of the CDI-III-PT scores.

	**Mean**	**Std. deviation**	**Minimum**	**Maximum**	**Skewness**	**Kurtosis**
Vocabulary—total	70.01	35.18	2.00	166.00	0.45	−0.35
Vocabulary—body parts	15.49	6.96	0.00	34.00	0.26	−0.27
Vocabulary—food	15.88	8.08	0.00	37.00	0.30	−0.47
Vocabulary—mental terms	20.17	12.07	0.00	45.00	0.24	−0.96
Vocabulary—emotions	18.46	11.02	0.00	50.00	0.84	0.32
Syntax	18.21	6.23	0.00	26.00	−0.54	−0.52

### Intercorrelations Among the CDI-III-PT Scores and the Scores in Other Language Measures

Table 4 presents the intercorrelations among the CDI-III-PT scores and the Griffiths language scores. The intercorrelations among the CDI-III-PT scores were positive and high. Moreover, medium-to-large correlations between the vocabulary and syntax scores obtained in the CDI-III-PT and the scores obtained in the Griffiths language subscale were obtained. Although non-significant, probably due to the low statistical power, the correlations between the scores in the Griffiths language subscale and the two first sections of the CDI-III-PT vocabulary subscale—body parts and food—were medium-sized.

**Table 4:** Correlations among the CDI-III-PT scores and the Griffiths scores.

	**Vocabulary—total**	**Vocabulary—body parts**	**Vocabulary—food**	**Vocabulary—mental terms**	**Vocabulary—emotions**	**Syntax**	**Griffiths—language score**
Vocabulary—total	1	0.884^***^	0.913^***^	0.949^***^	0.925^***^	0.659^***^	0.486^*^
Vocabulary—body parts		1	0.860^***^	0.767^***^	0.719^***^	0.583^***^	0.372
Vocabulary—food			1	0.804^***^	0.756^***^	0.598^***^	0.364
Vocabulary—mental terms				1	0.861^***^	0.646^***^	0.529^**^
Vocabulary—emotions					1	0.589^***^	0.520^*^
Syntax						1	0.445^*^
Griffiths—language score							1

*N = 23 for the correlations with the Griffiths scores; N = 739 for the remaining correlations*.

*** *p < 0.001;*

** *p < 0.01;*

* *p < 0.05*.

### Factors Associated With the CDI-III-PT Vocabulary and Syntax Scores

**Figure 1:**
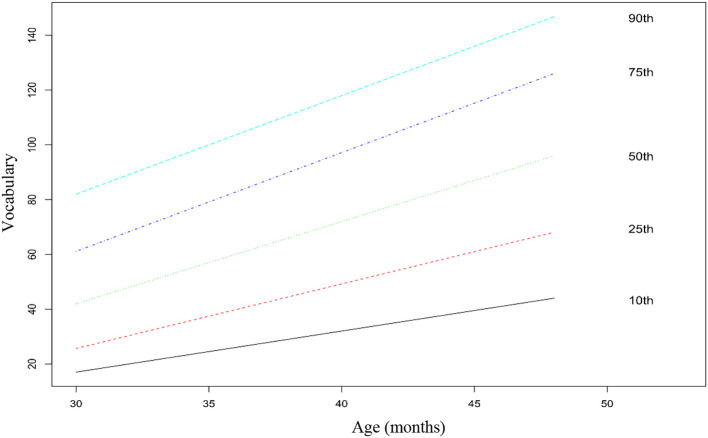
Vocabulary scores for percentiles 10th, 25th, 50th, 75th, and 90th across the age groups. Fitted by a linear model. *N* = 739.

**Figure 2:**
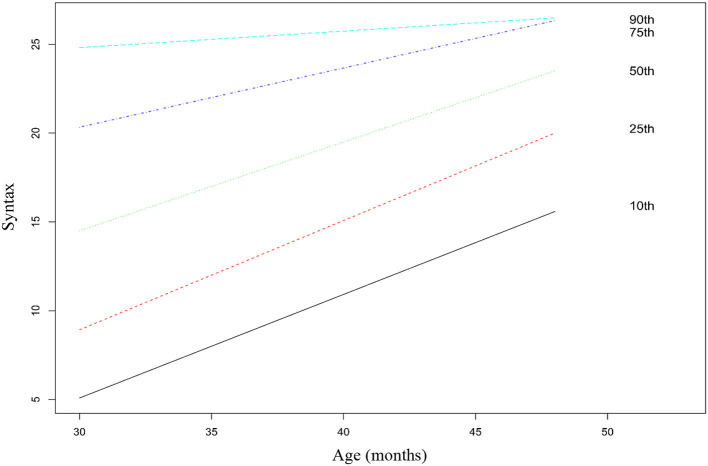
Syntax scores for percentiles 10th, 25th, 50th, 75th, and 90th across the age groups. Fitted by a linear model. *N* = 739.

Regarding multilevel analyses, the fully unconditional model had an intraclass correlation coefficient (ICC) of 0.052 for vocabulary and 0.068 for syntax, which indicates that 5 and 7% of the variance in the two subscales was associated with the preschool institutions attended by the children. Although this value is low, we proceeded with multilevel analyses to address the nested data structure. In a second step, we introduced the fixed individual predictors [age, gender, maternal education, and birth order (first born/later born)] in separate models for vocabulary and syntax. As expected, the models with the predictors had a significantly better fit than the fully unconditional models [vocabulary: $\chi_{\left(4\right)}^{2}$ = 488.08, *p* < 0.001; syntax: $\chi_{\left(4\right)}^{2}$ = 333.31, *p* < 0.001]. The results of these analyses are presented in Table 5. The model with the four predictors explained 13% and 10% of the variance in vocabulary and syntax, respectively. Maternal education was a significant predictor of both vocabulary and syntax. The results indicate that the children of mothers with a higher education degree have higher vocabulary and syntactic abilities than children of mothers with lower educational levels. The results also indicate that first born children have higher scores in vocabulary than later born children. No significant effect of birth order was found for syntax. Additionally, no significant effects were found for gender in any of the two subscales. Age was a significant predictor of both skills, after controlling for the effects of remaining variables, thus indicating that the CDI-III-PT scores capture the developmental changes in vocabulary and syntax that are expected with age. No ceiling or floor effects were found for the vocabulary subscale, as can be seen in the growth chart presented in Figure 1. Regarding the syntax subscale, the growth chart presented in Figure 2 suggests a ceiling effect for the 90th percentile: the slope is flatter than the remaining ones and children hit the ceiling at 38 months old. Percentile tables for each age group are available in Appendix C.

**Table 5:** Multilevel analyses of the factors associated with the CDI-III-PT measures.

	**Vocabulary**				**Syntax**			
**Model**	**Fully unconditional**		**Model with predictors**		**Fully unconditional**		**Model with predictors**	
	**Estimate**	**SE**	**Estimate**	**SE**	**Estimate**	**SE**	**Estimate**	**SE**
Intercept	69.75^***^	1.72	64.60^***^	2.61	18.15^***^	0.32	17.36^***^	0.48
Age			2.53^***^	0.26			0.46^***^	0.05
Gender			−4.02	2.43			−0.05	0.44
Maternal education			7.73^*^	2.49			1.12^*^	0.45
First born child			5.29^*^	2.48			0.33	0.45
Variance: individual level	1172.76^***^	63.16	1014.47^***^	56.17	36.20^***^	1.96	32.42^***^	1.80
Variance: group level	63.98^*^	29.71	14.36	18.28	2.67^*^	1.11	1.01	0.75
*R*^2^ individual level	–		0.13		–		0.10	
*R*^2^ group level	–		0.78		–		0.62	
Deviance	7348.49		6860.41		4785.21		4451.90	

*N = 702; SE, Standard error*.

*** *p < 0.001;*

* *p < 0.05*.

### Correlation and Agreement Between Parents' and Teachers' Scores

Table 6 presents the correlations between scores provided by parents and preschool teachers in the CDI-III-PT. The correlations between parents' and teachers' scores for the same subscales were high, ranging between 0.536 and 0.662, suggesting that both informants rank children similarly. Table 7 presents the results of the paired samples *t*-test to test for mean differences in the scores of the two types of raters. Regarding vocabulary, no differences were found in the total score, body parts, and food and related terms, but a small difference was found for mental terms, and a medium-sized difference was found for emotions. A medium sized difference was also found in the syntax subscale. In all cases, parents reported higher scores than teachers.

**Table 6:** Pearson correlation coefficients between the CDI-III-PT scores collected from parents and from preschool teachers.

	**Parental reports**					
	**Vocabulary—total**	**Vocabulary—body parts**	**Vocabulary—food**	**Vocabulary—mental terms**	**Vocabulary—emotions**	**Syntax**
Preschool teachers' reports						
Vocabulary—total	0.643**	0.649^**^	0.615^**^	0.631^**^	0.522^*^	0.494^*^
Vocabulary—body parts	0.621^**^	0.625**	0.578^**^	0.611^**^	0.517^*^	0.478^*^
Vocabulary—food	0.555^**^	0.556^**^	0.536**	0.538^**^	0.457^*^	0.448^*^
Vocabulary—mental terms	0.665^**^	0.701^***^	0.646^**^	0.662**	0.502^*^	0.487^*^
Vocabulary—emotions	0.660^**^	0.626^**^	0.620^**^	0.649^**^	0.573**	0.506^*^
Syntax	0.543^**^	0.537^**^	0.570^**^	0.514^*^	0.433^*^	0.609**

*N = 23*.

*** *p < 0.001;*

** *p < 0.01;*

* *p < 0.05. The values in bold are the correlations between informants for the same subscales*.

**Table 7:** Mean differences between the CDI-III-PT scores collected from parents and from preschool teachers.

**Variable**	**Mean**	**Std. deviation**	***t*_**(22)**_**	***p***	**Cohen's *d***
Vocabulary—total					
Parental reports	69.43	37.90	1.802	0.085	0.38
Preschool teachers' reports	56.96	40.46			
Vocabulary—body parts					
Parental reports	14.39	7.86	0.222	0.827	0.05
Preschool teachers' reports	14.04	9.27			
Vocabulary—food					
Parental reports	15.26	8.79	1.024	0.317	0.21
Preschool teachers' reports	13.26	10.43			
Vocabulary—mental terms					
Parental reports	20.74	12.82	2.179	0.040	0.45
Preschool teachers' reports	16.04	12.26			
Vocabulary—emotions					
Parental reports	19.04	11.03	2.694	0.013	0.56
Preschool teachers' reports	13.70	9.29			
Syntax					
Parental reports	17.78	5.59	2.494	0.021	0.52
Preschool teachers' reports	14.83	6.97			

## Discussion

This study addresses the psychometric properties of the European Portuguese adaptation of the CDI-III (CDI-III-PT), a parental report measure developed to assess expressive vocabulary and syntactic abilities of children aged between 2;6 and 4;0.

The internal consistency of the scores was high for both subscales. Moreover, the effect of age on the CDI-III-PT scores suggests sensitivity to the developmental changes of the measured abilities. No ceiling effects were found for the vocabulary subscale, but a ceiling effect on the syntax subscale was found. Ceiling effects on the syntax subscales had already been found in other CDI-III versions (Fenson et al., 2007; Eriksson, 2017), although there are variations in the composition of the subscales among the different versions. However, this ceiling effect is observed here only in the upper percentile (90th), which suggests that the measure will be more discriminative in children with average or poor syntactic skills. Therefore, the measure can be useful to perform a quick screening of protracted development in children's expressive language, supporting the referencing of the flagged children to a more comprehensive assessment of their linguistic and cognitive abilities.

Evidence of validity based on test-criterion relationships was collected by analyzing the effects of the child's gender, maternal education, and birth order on the CDI-III-PT scores. No gender differences were found in any of the two subscales, supporting the findings of other studies that suggest that boys catch up to girls' language abilities in preschool years (Altinkaynak, 2019; Fung et al., 2019; Jin et al., 2020; van der Wilt et al., 2020). Significant effects were found for maternal education in both subscales, a result which is also congruent with the findings of previous research in early development (Umek et al., 2006; Weigel et al., 2006; Fenson et al., 2007; Richels et al., 2013; Andonova, 2015; Gonzalez et al., 2017; Cadime et al., 2018; Farley and Piasta, 2020). Regarding birth order, a significant effect was found in vocabulary, indicating that the advantage in lexical abilities of first born children which has been found in toddlers (Fenson et al., 1994; Hoff-Ginsberg, 1998; Berglund et al., 2005; Zambrana et al., 2012) is maintained at least until the fourth year of age. However, an advantage was not found in syntactic abilities. It is unclear whether this result is due to the ceiling effect found for this subscale. Future studies should address if there is an effect of birth order in Portuguese-speaking preschoolers, using additional syntax measures.

A large correlation between the vocabulary and syntax scores of the CDI-III-PT was found, similarly to the ones obtained in other CDI-III versions (Eriksson, 2017; Tulviste and Schults, 2020). When correlations between the syntax scale and each of the different sections of the vocabulary scale are considered, higher correlation is found between the syntax scale and the section Vocabulary—mental terms. This is an expected result, given the relevance of mental state verbs included in the vocabulary scale to syntactic development in this age period, namely in what concerns complement clauses. A few verbs included in this section of the vocabulary scale even overlapped the set of verbs occurring with complement clauses in the syntax scale (e.g., *querer* “want,” *achar* “think,” *prometer* “promise”).

Regarding the evidence of convergent validity, medium-to-large correlations between the CDI-III-PT scores and the ones obtained in a standardized measure of children's language were obtained. These findings provide evidence of this type of validity for the scores obtained with the Portuguese version.

The results of this study also indicate high correlations between the reports of parents and teachers, thus suggesting a strong association between the scores from the two types of caregivers. The risk of subjectivity has been pointed out as a limitation of parental reports, although research has shown that this risk is minimized by using checklists of behaviors that can easily be observed and that do not require a retrospective report (Thal et al., 2000; Fenson et al., 2007). The high correlation between different informants suggests that the content of the CDI-III-PT is comprehensible and unambiguous, even for informants without a deep knowledge about children's language development, and that the items are clearly recognizable in daily contact with the children. However, when analyzing the mean scores, we found significantly higher mean scores in parents' reports, particularly in the section of emotions in the vocabulary checklist and in the syntax subscale. Although a difference was also found in the section of mental terms, the effect size was small. The reasons for these differences are yet to be clarified. On the one hand, this finding may indicate that the parents overestimate children's linguistic abilities compared to teachers, or, on the contrary, it may indicate that teachers underestimate them, given that they deal with a large group of children and may not be as much aware of children's productions as parents. On the other hand, these mean differences between the two types of informants may simply reflect the contextual nature of language. For example, it is plausible that children use more emotion words at home and use them less at daycare/preschool. Future studies should address these issues.

As a final remark, we would highlight that the CDI-III-PT is not a mere translation of the versions developed for other languages. Instead, a major concern was that the test content accurately mirrored the structures expected to occur in the spontaneous production of European Portuguese-speaking children. In the case of the vocabulary subscale, the structure adopted is similar to the one used in other CDI-III versions (Swedish and Estonian; Eriksson, 2017; Tulviste and Schults, 2020), but the words that composed it were mainly retrieved from other measures and databases for European Portuguese (Santos et al., 2016a; Silva et al., 2017b). In the case of the syntax subscale, the structures expected for this age interval were carefully considered and the items were developed in order to represent them, as indicated in the description of the measures. The application of these procedures during the development of the test aimed to increase the validity of the scores, namely the validity based on test content [American Educational Research Association (AERA) et al., 1999].

The main limitation of the present study was the low sample size of the two sub-samples used to study the relation between the CDI-III-PT scores and the scores of a standardized measure of language and to study the relation between the scores obtained from parents and from preschool teachers. Future studies should investigate these relationships using a larger and more representative sample. Another limitation is that, due to the ceiling effect in the upper percentiles, the syntax subscale will not be very discriminant when assessing children with very high levels of syntactic abilities. Thus, this subscale is more appropriate to assess children with low or average syntactic abilities.

In conclusion, the CDI-III-PT is an instrument with adequate psychometric properties that can be a useful tool for research and practice. Given that it is a short measure (2 pages), it can be used to collect information from large samples in a short period of time, which is very cost-effective for research. Short report measures are also less prone to withdrawal in research. Moreover, the items of both subscales can be individually analyzed to qualitatively explore the acquisition of specific syntactic structures or specific words in European Portuguese. The similarity of the vocabulary subscale with the ones of the CDI-III versions recently developed for other languages, such as Swedish and Estonian, make it appropriate for cross-linguistic research. As previously stated, the syntax subscale has a particular format and was designed to accommodate the specificities of European Portuguese, but the represented structures can also be individually analyzed and the results can be qualitatively compared to children's productions in other languages.

Regarding the applications in practice, namely clinical practice, the CDI-III-PT can be used as a tool to provide a quick overview of a child's expressive language in comparison with their peers using the age-based norms for the Portuguese population. Further research is under development to address the establishment of a cutoff point in the scores to flag children with language difficulties that should be referenced to a more complete assessment, given that these difficulties can be a manifestation of a more permanent impairment.

## Data Availability Statement

The raw data supporting the conclusions of this article will be made available by the authors, without undue reservation.

## Ethics Statement

The studies involving human participants were reviewed and approved by the Portuguese Commission of Data Protection. Written informed consent to participate in this study was provided by the participants' legal guardian/next of kin.

## Author Contributions

IC made substantial contributions to the development of the test materials, conception and design of the study, data collection, statistical data analysis and interpretation, and discussion of the results. AS made substantial contributions to the development of the test materials, conception and design of the study, data collection and interpretation, and discussion of the results. FV and IR made substantial contributions to the conception and design of the study and interpretation and discussion of the results. All authors were involved in drafting the manuscript and revising it critically for important intellectual content.

## Conflict of Interest

The authors declare that the research was conducted in the absence of any commercial or financial relationships that could be construed as a potential conflict of interest.
