# Supplementary material for: Parental Reports of Preschoolers' Lexical and Syntactic Development: Validation of the CDI-III for European Portuguese
Source: Front Psychol. 2021 Jul 22;12:677575. doi: 10.3389/fpsyg.2021.677575 (PMC8344901; doi:10.3389/fpsyg.2021.677575)
Supplement: Supplementary file 1 [file Data_Sheet_1.docx]

Supplementary Material

**APPENDIX A**

**Inventário do Desenvolvimento de Habilidades Comunicativas MacArthur-Bates – Versão em Português Europeu**

**2 anos e 6 meses – 4 anos e 0 meses**

**(Cadime, Santos, Ribeiro, & Viana, 2021)**

**VOCABULÁRIO**

Por favor, assinale as palavras que **já ouviu a criança dizer** no seu quotidiano (**não nos interessa** aquilo que a criança é capaz de repetir ou imitar, mas apenas as palavras que a criança usa espontaneamente quando fala). Assinale a palavra mesmo que a sua pronúncia não seja perfeita (por exemplo, se diz *meixa* em vez de ameixa). No caso dos verbos, assinale o verbo independentemente do tempo verbal e da pessoa utilizada (por exemplo, assinale o verbo “achar” se a criança diz “eu acho”, “eu achei”, “tu achas”, ou outra forma do mesmo verbo). Tenha em conta que esta é uma lista de palavras que podem ser usadas por crianças de diferentes idades, pelo que é normal que o seu filho diga apenas algumas palavras da lista.

**Partes do corpo e palavras relacionadas**

| *Nomes* | **Diz** | *Nomes* | **Diz** | *Nomes* | **Diz** | *Nomes* | **Diz** | *Nomes* | **Diz** | *Verbos e adj.* | **Diz** | *Verbos e adj.* | **Diz** |
| --- | --- | --- | --- | --- | --- | --- | --- | --- | --- | --- | --- | --- | --- |
| Cérebro | ⬜ | Febre | ⬜ | Lábios | ⬜ | Pestana | ⬜ | Testa | ⬜ | Assoar | ⬜ | Transpirado(a) | ⬜ |
| Coração | ⬜ | Ferida | ⬜ | Mindinho | ⬜ | Polegar | ⬜ | Tornozelo | ⬜ | Doente | ⬜ | Suado(a) | ⬜ |
| Costas | ⬜ | Fígado | ⬜ | Muco/ranho | ⬜ | Pulmão(ões) | ⬜ |  |  | Doer | ⬜ |  |  |
| Cotovelo | ⬜ | Garganta | ⬜ | Ombros | ⬜ | Queixo | ⬜ |  |  | Sarar | ⬜ |  |  |
| Esqueleto | ⬜ | Gengiva | ⬜ | Osso(s) | ⬜ | Sangue | ⬜ |  |  | Saudável | ⬜ |  |  |
| Estômago | ⬜ | Joelho | ⬜ | Peito | ⬜ | Sobrancelha | ⬜ |  |  | Tossir | ⬜ |  |  |

**Comida e palavras relacionadas**

| *Nomes* | **Diz** | *Nomes* | **Diz** | *Verbos* | **Diz** | *Verbos* | **Diz** | *Verbos* | **Diz** | *Verbos* | **Diz** | *Verbos* | **Diz** |
| --- | --- | --- | --- | --- | --- | --- | --- | --- | --- | --- | --- | --- | --- |
| Ameixa | ⬜ | Sal | ⬜ | Acender | ⬜ | Cheirar | ⬜ | Envolver | ⬜ | Fritar | ⬜ | Queimar | ⬜ |
| Amêndoa | ⬜ |  |  | Adicionar | ⬜ | Cortar | ⬜ | Enxaguar | ⬜ | Juntar | ⬜ | Saborear | ⬜ |
| Avental | ⬜ |  |  | Aquecer | ⬜ | Cozer | ⬜ | Escorrer | ⬜ | Limpar | ⬜ | Servir | ⬜ |
| Chá | ⬜ |  |  | Arrefecer | ⬜ | Descascar | ⬜ | Esmagar | ⬜ | Mexer | ⬜ | Temperar | ⬜ |
| Forno | ⬜ |  |  | Assar | ⬜ | Encher | ⬜ | Estufar | ⬜ | Misturar | ⬜ | Torrar | ⬜ |
| Lata | ⬜ |  |  | Barrar | ⬜ | Entornar | ⬜ | Ferver | ⬜ | Provar | ⬜ | Verter | ⬜ |

**Termos cujo significado se relaciona com (ou envolve) processos mentais**

| *Nomes* | **Diz** | *Verbos e adj.* | **Diz** | *Verbos e adj.* | **Diz** | *Verbos e adj.* | **Diz** | *Verbos e adj.* | **Diz** | *Verbos e adj.* | **Diz** |
| --- | --- | --- | --- | --- | --- | --- | --- | --- | --- | --- | --- |
| Dúvida | ⬜ | Achar | ⬜ | Dever | ⬜ | Fingir | ⬜ | Perceber | ⬜ | Recordar | ⬜ |
| Ideia | ⬜ | Acreditar | ⬜ | Duvidar | ⬜ | Imaginar | ⬜ | Permitir | ⬜ | Relembrar | ⬜ |
| Pesadelo | ⬜ | Adivinhar | ⬜ | Enganar | ⬜ | Inventar | ⬜ | Poder | ⬜ | Saber | ⬜ |
|  |  | Apetecer | ⬜ | Ensinar | ⬜ | Lembrar | ⬜ | Precisar | ⬜ | Sentir | ⬜ |
|  |  | Aprender | ⬜ | Esperto(a) | ⬜ | Mandar | ⬜ | Preferir | ⬜ | Sonhar | ⬜ |
|  |  | Compreender | ⬜ | Esquecer (-se) | ⬜ | Mentir | ⬜ | Procurar | ⬜ | Tentar | ⬜ |
|  |  | Conseguir | ⬜ | Estranho | ⬜ | Parecer | ⬜ | Proibir | ⬜ |  |  |
|  |  | Descobrir | ⬜ | Explicar | ⬜ | Pedir | ⬜ | Prometer | ⬜ |  |  |
|  |  | Desejar | ⬜ | Fantasiar | ⬜ | Pensar | ⬜ | Querer | ⬜ |  |  |

**Emoções e palavras relacionadas**

| *Nomes* | **Diz** | *Verbos e adj.* | **Diz** | *Verbos e adj.* | **Diz** | *Verbos e adj.* | **Diz** | *Verbos e adj.* | **Diz** | *Verbos e adj.* | **Diz** |
| --- | --- | --- | --- | --- | --- | --- | --- | --- | --- | --- | --- |
| Alegria | ⬜ | Aborrecido(a) | ⬜ | Atrevido(a) | ⬜ | Envergonhado(a) | ⬜ | Medroso(a) | ⬜ | Seguro(a) | ⬜ |
| Chatice | ⬜ | Abraçar | ⬜ | Cansado(a) | ⬜ | Esfomeado(a) | ⬜ | Nervoso(a) | ⬜ | Simpático(a) | ⬜ |
| Coragem | ⬜ | Adorar | ⬜ | Contente | ⬜ | Espantado(a) | ⬜ | Nojento(a) | ⬜ | Sossegado(a) | ⬜ |
| Maldade | ⬜ | Alegre | ⬜ | Corajoso(a) | ⬜ | Feliz | ⬜ | Odiar | ⬜ | Surpreendido(a) | ⬜ |
| Maluquice | ⬜ | Amar | ⬜ | Curioso(a) | ⬜ | Fofo(a) | ⬜ | Orgulhoso(a) | ⬜ | Triste | ⬜ |
| Medo | ⬜ | Apavorado(a) | ⬜ | Detestar | ⬜ | Furioso(a) | ⬜ | Parvo(a) | ⬜ |  |  |
| Nojo | ⬜ | Arrependido(a) | ⬜ | Difícil | ⬜ | Infeliz | ⬜ | Perigoso(a) | ⬜ |  |  |
| Orgulho | ⬜ | Assustado(a) | ⬜ | Engraçado(a) | ⬜ | Irritado(a) | ⬜ | Querido(a) | ⬜ |  |  |
| Raiva | ⬜ | Assustar | ⬜ | Enjoado(a) | ⬜ | Malandro(a) | ⬜ | Satisfeito(a) | ⬜ |  |  |

**FRASES**

Por favor, assinale na lista seguinte o tipo de frases que a criança produz no seu quotidiano. As frases podem ser muito diferentes das apresentadas. O que pretendemos é saber **se as crianças usam as palavras sublinhadas** em **frases semelhantes** às que são apresentadas.

Tome como exemplo o item 2. Assinale *Diz,* se o seu filho diz “O cão quer fazer chichi” ou “O bebé quer beber água”, ou “o bebé quer sair”, ou ainda outros exemplos que não estejam contemplados, como por exemplo “A Ana quer ir à praia”. Neste exemplo, o importante é reconhecer se o seu filho utiliza o verbo *querer* (sublinhado), seguido por outro verbo no infinitivo (terminação sublinhada). O mesmo deve ser feito para as restantes frases.

|  | **Não diz** | **Diz** |
| --- | --- | --- |
| 1. O bebé **quer** papinha. / O cão **quer** o osso. | ⬜ | ⬜ |
| 1. O cão **quer** faz**er** chichi. / O bebé **quer** beb**er** água. / O bebé **quer** sa**ir**. | ⬜ | ⬜ |
| 1. O bebé **queria** **que** a mãe empurr**asse** o carro. | ⬜ | ⬜ |
| 1. O cão **pode** sa**ir**. / O bebé **pode** com**er** a papa. | ⬜ | ⬜ |
| 1. Eu **gosto de** v**er** as formigas. / Ele **gosta de** and**ar** de baloiço / Tu **gostas de** corr**er**. | ⬜ | ⬜ |
| 1. Eu **acho que sim**/ Eu **acho que não** / Tu **achas que sim**. | ⬜ | ⬜ |
| 1. Eu **acho que** é um menino. / **Acho que** ele gosta. / **Acho que** foi embora. | ⬜ | ⬜ |
| 1. **Deixa-me** ver (ou deixa ver) / Ele **deixou** cair o copo. | ⬜ | ⬜ |
| 1. A mãe **disse que** comprou um gelado/ O mano **disse que** ia à rua. | ⬜ | ⬜ |
| 1. **Vi** o cão **a** faz**er** cocó. / **Viste** o passarinho **a** vo**ar**? / Ele **viu** a menina **a** com**er**. | ⬜ | ⬜ |
| 1. **Ajuda** a Rita **a** faz**er** o desenho. / **Ajuda** a mana **a** pint**ar** o porco/ O pai **ajudou** o menino **a** com**er**. | ⬜ | ⬜ |
| 1. **Ensina-me** **a** pint**ar** o desenho. / **Ensina** o mano **a** jog**ar**. | ⬜ | ⬜ |
| 1. Ela **pôs** o bebé **a** dorm**ir**. / Ele **pôs** a menina **a** com**er**. | ⬜ | ⬜ |
| 1. A mãe **prometeu** **ao** menino faz**er** o desenho. | ⬜ | ⬜ |
| 1. **O que** é**?** / **Que** é**?** / **O que** foi**?** / **Que** foi**?** | ⬜ | ⬜ |
| 1. Quem **é que** deu**?** / Quem **é que** fez**?** / O que **é que** queres**?** | ⬜ | ⬜ |
| 1. **Onde** **é que** o pai pôs a bola? | ⬜ | ⬜ |
| 1. **Porque** **é que** a avó não sabe conduzir? | ⬜ | ⬜ |
| 1. **Como** **é que** o gato foi para ali? | ⬜ | ⬜ |
| 1. Encontrei o boneco **que** o mano estragou. / Vi o gato **que** o cão mordeu. | ⬜ | ⬜ |
| 1. A casa **onde** eu moro é bonita. / O lugar **onde** deixei a boneca está sujo. | ⬜ | ⬜ |
| 1. A mãe **é que** dá banho. / O João **é que** foi passear. | ⬜ | ⬜ |
| 1. **Foi** o mano **que** riscou a parede. / **É** a avó **que** dá chocolate. | ⬜ | ⬜ |
| 1. **Quem** riscou a parede **foi** o mano. / **Quem** dá chocolate **é** a avó. | ⬜ | ⬜ |
| 1. A mãe vai fazer o jantar **quando** chegar a casa. / **Quando** o pai chegar vamos à rua. | ⬜ | ⬜ |
| 1. **Se** vais a correr, podes cair. / A mãe não dá colo **se** chorares. / **Se** fizeres birra, a mãe não dá colo. | ⬜ | ⬜ |

**APPENDIX B**

Table A. *Curve estimation: model fit for the growth models*

|  | R^2^ | R^2^ Adjusted | Sum of squares | F (df) | p |
| --- | --- | --- | --- | --- | --- |
| Vocabulary |  |  |  |  |  |
| Linear | .125 | .124 | 114325.951 | 105.482 (1, 737) | <.001 |
| Quadratic | .126 | .124 | 114947.460 | 52.997 (2, 736) | <.001 |
| Cubic | .126 | .124 | 115043.522 | 53.047 (2, 736) | <.001 |
| Syntax |  |  |  |  |  |
| Linear | .123 | .122 | 3513.254 | 103.200 (1, 737) | <.001 |
| Quadratic | .124 | .121 | 3541.191 | 51.998 (2, 736) | <.001 |
| Cubic | .124 | .121 | 3541.621 | 52.005 (2, 736) | <.001 |


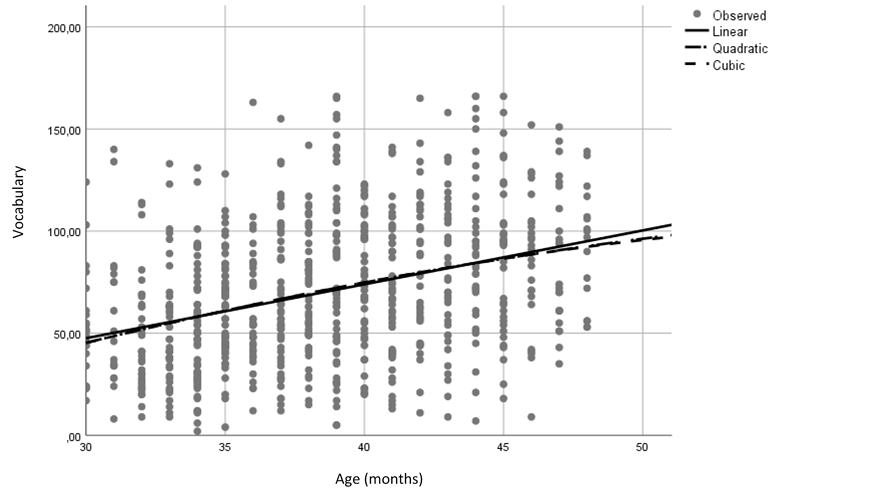


Figure A. *Vocabulary: fit lines for linear, quadratic and cubic growth models*


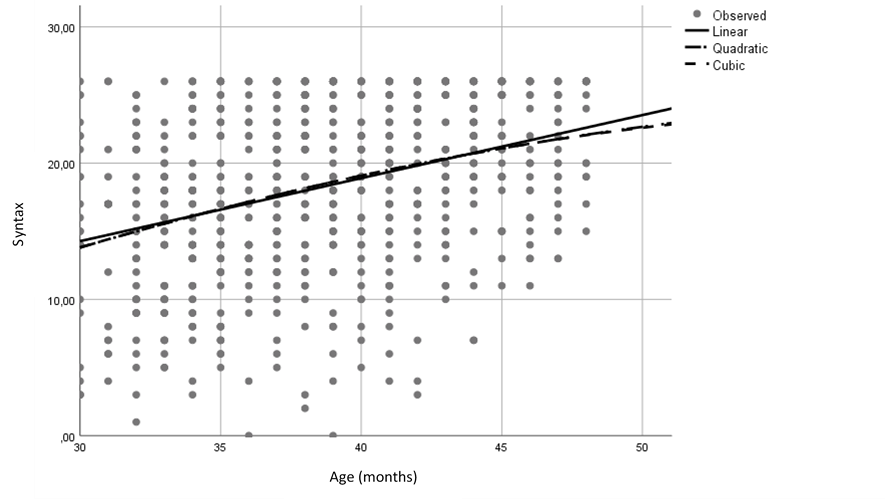


Figure B. *Syntax: fit lines for linear, quadratic and cubic growth models*

**APPENDIX C**

Table A. *Percentiles for the vocabulary subscale of the CDI-III-PT.*

|  |  | Percentile | | | | |
| --- | --- | --- | --- | --- | --- | --- |
| Age (months) | N | 10th | 25th | 50th | 75th | 90th |
| 30 | 24 | 17 | 26 | 42 | 61 | 82 |
| 31 | 18 | 19 | 28 | 45 | 65 | 86 |
| 32 | 38 | 20 | 30 | 48 | 68 | 89 |
| 33 | 40 | 22 | 33 | 51 | 72 | 93 |
| 34 | 56 | 23 | 35 | 54 | 76 | 96 |
| 35 | 58 | 25 | 37 | 57 | 79 | 100 |
| 36 | 37 | 26 | 40 | 60 | 83 | 104 |
| 37 | 51 | 28 | 42 | 63 | 86 | 107 |
| 38 | 56 | 29 | 45 | 66 | 90 | 111 |
| 39 | 60 | 31 | 47 | 69 | 94 | 114 |
| 40 | 44 | 32 | 49 | 72 | 97 | 118 |
| 41 | 49 | 34 | 52 | 75 | 101 | 122 |
| 42 | 37 | 35 | 54 | 78 | 104 | 125 |
| 43 | 37 | 37 | 56 | 81 | 108 | 129 |
| 44 | 37 | 38 | 59 | 84 | 112 | 132 |
| 45 | 33 | 40 | 61 | 87 | 115 | 136 |
| 46 | 25 | 41 | 63 | 90 | 119 | 140 |
| 47 | 23 | 43 | 66 | 93 | 122 | 143 |
| 48 | 16 | 44 | 68 | 96 | 126 | 147 |

*Note*: Values fitted by a linear function. N=739.

Table B. *Percentiles for the syntax subscale of the CDI-III-PT.*

|  |  | Percentile | | | | |
| --- | --- | --- | --- | --- | --- | --- |
| Age (months) | N | 10th | 25th | 50th | 75th | 90th |
| 30 | 24 | 5 | 9 | 15 | 20 | 25 |
| 31 | 18 | 6 | 10 | 15 | 21 | 25 |
| 32 | 38 | 6 | 10 | 16 | 21 | 25 |
| 33 | 40 | 7 | 11 | 16 | 21 | 25 |
| 34 | 56 | 7 | 11 | 17 | 22 | 25 |
| 35 | 58 | 8 | 12 | 17 | 22 | 25 |
| 36 | 37 | 9 | 13 | 18 | 22 | 25 |
| 37 | 51 | 9 | 13 | 18 | 23 | 25 |
| 38 | 56 | 10 | 14 | 19 | 23 | 26 |
| 39 | 60 | 10 | 14 | 19 | 23 | 26 |
| 40 | 44 | 11 | 15 | 20 | 24 | 26 |
| 41 | 49 | 12 | 16 | 20 | 24 | 26 |
| 42 | 37 | 12 | 16 | 21 | 24 | 26 |
| 43 | 37 | 13 | 17 | 21 | 25 | 26 |
| 44 | 37 | 13 | 18 | 22 | 25 | 26 |
| 45 | 33 | 14 | 18 | 22 | 25 | 26 |
| 46 | 25 | 14 | 19 | 23 | 26 | 26 |
| 47 | 23 | 15 | 19 | 23 | 26 | 26 |
| 48 | 16 | 16 | 20 | 24 | 26 | 26 |

*Note*: Values fitted by a linear function. N=739.
